# Neurocomputational training to boost reward learning and sensitivity in anhedonic individuals: a transdiagnostic proof-of-concept trial

**DOI:** 10.1038/s41398-026-04355-1

**Published:** 2026-08-03

**Authors:** Katia M. Harlé, Murray B. Stein, Alan N. Simmons, Charles T. Taylor

**Affiliations:** 1 VA San Diego Healthcare System, San Diego, CA USA; 2 Department of Psychiatry, UCSD, La Jolla, CA USA; 3 Center of Excellence in Stress and Mental Health, VA San Diego Healthcare System, San Diego, CA USA

**Keywords:** Depression, Neuroscience

## Abstract

Anhedonia, which is characterized by a loss of pleasure and engagement in rewarding activities, is prevalent across diagnostic groups and is associated with worse clinical and treatment outcomes. Existing evidence-based treatments have only modest effects on improving anhedonia. Reward learning (RL) is an important driver of reward-responsive behavior, which could be leveraged to improve reward sensitivity - a candidate mechanism underlying anhedonia. Individuals (N = 50) with at least moderate anhedonia and clinical levels of depression and/or anxiety were randomized to either an active RL training (high variance of reward rates to promote reward exploration and maximization) or a sham version. Before and after training, they completed an assessment RL task while undergoing functional neuroimaging as well as ratings of current affect. Computational modeling was used to assess change in reward learning and sensitivity, as well as associated neural activity. Participants assigned to the active condition exhibited greater increases in learning-based reward maximization, i.e., the propensity to choose options predicted to be most rewarding, and reductions in anterior cingulate cortex activation to reward prediction errors (discrepancies between expected and observed rewards), consistent with optimization of reward learning and performance. In exploratory analyses, learning-based reward maximization during training mediated increase in positive affect associated with the active condition. Our results underscore the potential of RL-informed computerized trainings to improve RL and associated goal-directed reward behavior in anhedonic individuals. Future research is needed to determine whether the proposed training can successfully transfer to real-world settings and produce long-term affective and clinical improvements (**ClinicalTrials.gov**: NCT05383248).

## Introduction

Anhedonia is a clinical phenotype characterized by blunted positive affect and reduced responsiveness to rewards, such as previously enjoyed hobbies, social connections, etc. [1,2]. This pathology is a common symptom of major depressive disorder (MDD; [3], and is also prominent across many psychiatric diagnoses, including anxiety disorders [4], post-traumatic stress disorder [5–7], and other severe mental illness [8–10]. Moreover, anhedonia is associated with poor psychosocial functioning [1,6,11,12], increased suicidal behaviors [13,14], and poorer responsiveness to pharmacological [15], neurostimulation [16], and psychotherapeutic [7,17,18] treatment.

Despite these adverse outcomes, anhedonia-focused interventions are scarce. Behavioral treatments such as cognitive behavioral therapy (CBT) remain only partially effective in treating anhedonia [19]. Most evidence-based treatments focus on modifying negative affect and punishment-driven symptoms (e.g., negative thinking, avoidance patterns) rather than targeting reward system mechanisms that are believed to underpin anhedonia [20–22]. One exception is behavioral activation (BA), which targets reward responsiveness by encouraging patients to schedule rewarding activities in their daily lives [23], as well as more recent positive affect/reward-centric protocols combining BA and a range of cognitive and mindfulness strategies, e.g., as Behavioral Activation Therapy for Anhedonia/BATA [24], Amplification of Positivity/AMP [25] and Positive Affect Treatment/PAT [26]. However, to implement BA, clinicians often face the challenge of low patient compliance, particularly in more severe depressive pathology, with limited ability to reduce anhedonia to normative healthy levels [19]. Thus, there is a strong need for new reward-focused treatment models for anhedonia. Mechanistically informed cognitive trainings that are scalable and portable (e.g., suitable for remote administration with minimal therapist involvement) could provide a more customizable and cost-effective therapeutic format to target reward processing more precisely and enhance the effectiveness of existing treatments.

While several neurocognitive components of the reward system have been implicated in hedonic function [27–30], reward learning (RL) is a key mechanism implicated in hedonic function. Instrumental RL is a goal-directed, top-down process, which engages executive processes, including explicit need to attend to, maintain, and manipulate specific rules guiding behavior (e.g., reward maximization) and choice-outcomes contingencies. Thus, in contrast to interventions targeting implicit learning or automatic affective processing, this type of intervention may specifically promote strengthening of top-down control, which is critical to support goal-directed reward behavior. RL involves the development of predictions of stimulus-reward contingencies and the integration of such predictions into decision-making [31], and is therefore a potentially important therapeutic target for training the brain to seek healthy rewards. Computational models of adaptive RL have provided a more precise understanding of reward learning and how this function may be altered in mental illness [31–33]. These models highlight two relatively independent computational challenges: (1) the development of reward predictions and their dynamic updating based on repeated sampling from choice options (exploration) and observation of outcomes [31], and (2) the decision strategies whereby ones selects options, e.g., learning-based reward maximization [34–36]. Representation and updating of such internal predictions in the brain are further facilitated via reward prediction errors (RPE), i.e., discrepancies between obtained and expected reward, which have been robustly observed in the ventral striatum (VS), midbrain/thalamus, and medial prefrontal cortex, particularly the dorsal anterior cingulate cortex (ACC; [37,38]). Critically, anhedonia has been linked to blunted computational indices of learning-based reward maximization, i.e., reduced strategic tendency to choose options predicted to be most rewarding, which may mediate behavior observed in depressed individuals [31,34,35,39,40]. In terms of neural processes, blunted striatal RPE signals are associated with depression [40,41], while anhedonia has been linked to increased RPE signals in the ACC [42]. Greater RPE signals in the ACC at baseline also predicts greater reduction in anhedonia and quality of life in individuals with mood disorders [43]. Moreover, anhedonic depression is associated with slower, less efficient reward learning [41], which could explain findings of larger RPE signals [42] if predictions are less accurate [27,44]. Thus, RL plays a key role in goal-directed reward exploration and maximization, and is likely to play a role in modulating anhedonia, which involves deficits in reward seeking-behavior. Yet, reinforcement learning remains unexplored as a cognitive training therapeutic target to boost reward sensitivity and reward-responsive behavior.

Thus, the goal of this study was to test whether a classic RL paradigm, the multi-arm bandit (MAB) task [35,45–47], could be leveraged to boost reward learning and reward maximizing behavior among anhedonic individuals. Reward variance has been shown to play a critical role in boosting reward exploration and maximization. Both the distinctiveness of available options (span of underlying reward rates) and temporal shifts in reward rates increase the salience of these options and drive approach behavior [47–50]. In the MAB paradigm, in which one has to explore and sample from different options with unknown reward rates with the goal of maximizing rewards, higher variance of reward rates is associated with larger learning-based reward maximization [51]. We developed a MAB-based cognitive training tuned to provide exploration of a high reward variance environment (active) to be compared with a low-variance condition (sham). In a 2-arm randomized clinical trial (RCT), this study aimed to test whether such active RL training was effective in shifting behavioral and neural markers of reward learning and sensitivity (assessed with RL computational modeling) among individuals with at least moderate anhedonia. We predicted that, relative to sham, the active condition would lead to greater increases in learning-based reward maximization. At the neural level, based on the above research linking anhedonia and depression to abnormal RPE signals in the ACC and striatum, and given the significant role of these regions in integrating these surprise signals to update reward predictions and guide the degree of reward exploration vs reward maximizing behavior [52], we expected greater changes in RPE activations in these regions in the active group. These behavioral and neural hypotheses were included in pre-registration of this study. Finally, we aimed to explore whether targeting such neurocognitive markers could translate to shifts in positive affect.

## Methods

### Procedure

Participants were randomized 1:1 to one of two training conditions of a RL training, i.e., active (high-variance) or sham (low-variance; see below for details). All participants and all research staff administering the procedures were blinded to the condition allocation. Participants were assigned to one of the two conditions using a computer-generated blocked randomization sequence with coded labels (R:*blockrand*), a process which was handled by an auxiliary researcher who did not administer participant-facing procedures. Active and sham training tasks were identical in every aspect except for experimental settings of underlying reward outcome probabilities, e.g., instructions and any other training-related procedure did not differ between condition arms.

Immediately before and after completing the training task, participants completed a brief measure of state positive and negative affect (the Positive and Negative Affect Schedule/PANAS) [53] and an RL assessment task (see below). Participants completed the RL assessments while undergoing functional magnetic resonance imaging (fMRI). Prior to this, participants also completed self-report affective measures, including the Mood and Anxiety Symptoms Questionnaire (MASQ) [54], and were administered the Mini International Neuropsychiatric Interview for DSM-5 [55] to assess for psychiatric diagnoses. All procedures were approved by the UCSD Institutional Review Board and Human Research Protections Program. Participants received a payment of $100 for participating in the study, which included an MRI scan. The study followed the Declaration of Helsinki of 2013 and was preregistered (NCT05383248).

### Participants

Participants (N = 50) were treatment-seeking individuals with at least moderate anhedonia and clinical levels of depression and/or anxiety, recruited through posted announcements in community and online settings (e.g., Reddit). All participants consented to all study procedures. Inclusion criteria were: (1) age 18 to 65; (2) clinically significant levels of anhedonia/low positive affect (>7 on the Snaith-Hamilton Pleasure Scale/SHAPS, reverse-coded for anhedonia) [56] and/or <25 on PANAS-Positive subscale (probing positive affect over the past few weeks) [57]), equivalent to 1SD below normative population mean [26,53]; and (3) clinically elevated symptoms of anxiety (≥10 on a measure of anxiety, i.e., GAD-7 [58]) or depression (≥10 on the Patient Health Questionnaire-9/PHQ-9 [59]; see Supplementary Material for Exclusion Criteria).

Treatment groups did not significantly differ in terms of age, sex, race, ethnicity, or education (see Table 1). All participants had a principal diagnosis of MDD, Generalized Anxiety Disorder (GAD), or both. Overall, both groups also had similar diagnostic and clinical severity profiles at baseline, however, despite randomization, the active group had a higher proportion of individuals meeting diagnostic criteria for MDD (p = 0.02; Table 1). As a result, this variable was included as a covariate in the analyses.

**Table 1:** Demographic and clinical characteristics.

Measure	Active (N = 25)		Sham (N = 25)		p	Total (N = 50)	
	Mean	SD	Mean	SD		Mean	SD
**Demographics**							
Age	27.48	10.41	29.08	9.73	0.42	28.28	10.02
Sex (Female)	60%		52%		0.25	56%	
Ethnicity (Not Hispanic)	56%		74%		0.19	65%	
Race	40% White, 40% Asian, 20% Mixed		61% White, 26% Asian, 13% Mixed		0.39	51% White, 33% Asian, 16% Mixed	
Education (AA or higher)	79%		76%		0.79	78%	
**Diagnostic Profile (Axis I)**							
Major Depressive Disorder (MDD) Dx	76%		44%		0.02	60%	
Generalized Anxiety Disorder (GAD) Dx	64%		48%		0.25	56%	
Social Anxiety Disorder (SAD) Dx	52%		44%		0.57	48%	
Post-Traumatic Stress Disorder (PTSD) Dx	28%		28%		1.00	28%	
**Affective Profile**							
MASQ-General Distress Anxiety	30.12	7.49	27.08	7.72	0.17	28.63	7.68
MASQ-General Distress Depression	42.12	10.69	37.8	9.85	0.14	39.96	10.41
MASQ-Anxious Arousal	32.12	10.40	26.08	8.51	0.06	29.46	9.79
MASQ-Anhedonic Depression	83.12	13.58	81.92	11.29	0.74	82.52	12.36
**State Affect (Pre-Training)**							
PANAS – Positive Affect	20.79	7.71	19.00	6.56	0.29	19.89	7.13
PANAS – Negative Affect	18.04	7.04	15.08	4.62	0.11	16.53	6.06
**State Affect (Post-Training)**							
PANAS – Positive Affect	22.00	6.45	17.79	5.29	0.02	19.89	6.21
PANAS – Negative Affect	13.17	3.14	11.52	2.62	0.06	12.32	2.97

Axis I diagnoses reported in this table were measured with the Mini International Neuropsychiatric Interview for DSM-5;

*PANAS* positive and negative affect schedule (state affect), *MASQ* mood and anxiety symptoms questionnaire, *AA* associates degree, *Dx* diagnosis, Chi-square test of independence was used for categorical variables; Welch t-tests (for normally distributed data) or Mann-Whitney U tests (for non-normally distributed data) were used as appropriate; all *p* values indicate two-tailed statistical significance levels.

### Interventions

**Fig. 1:**
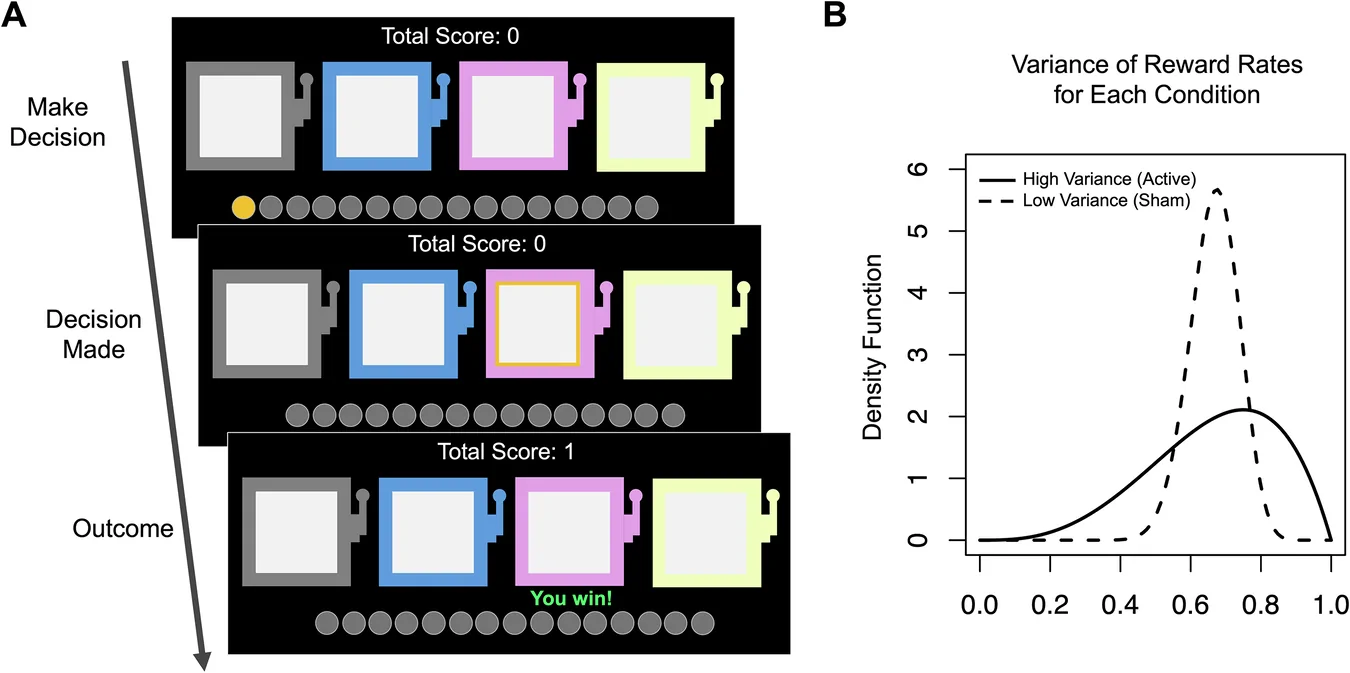
Reward Learning Training. **A** Trial Timeline (Reward Outcome Example). At the start of each trial, one of 16 tokens becomes available, which can be used to select one of 4 slot machines with unknown reward rates, with the goal to maximize reward in each game. After selecting one option, feedback is presented below chosen option, either “You win or “You didn’t win” in green or red, respectively. A win increases the game score by 1 pt, while a no-win outcome results in 0 pt earned. Reward rates underlying each arm changed at the beginning of each game. **B** Experimental Settings of Reward Rate Contingencies for each Condition. Both training conditions had a high-reward/abundant environment (mean reward = 0.67) but with different variance structures, i.e., drawn for each game from a high variance (Beta(α = 4, β = 2) or low variance (Beta(α = 30, β = 15) beta distribution for the active and sham condition, respectively. All participants saw the same sets of rates presented in randomized order within each respective condition.

In each training condition, participants completed 20 MAB blocks, each with 16 trials, in which they were instructed to explore 4 options with unknown reward rates (appearing as slot machines of different color) with the goal of maximizing overall rewards (points). On each trial, participants used one token to select an option, after which participants either earned 1 point and saw a ‘win’ feedback in green under the chosen option, or earned zero points and saw a ‘no-win’ feedback appearing in grey (Fig. 1A). For the active condition (high-variance), underlying reward rates were independently drawn for each block from a high reward/high variance beta distribution (i.e., Beta(α = 4, β = 2), mean = 0.67; SD = 0.18). This high-variance environment was used given evidence that both within-game variance (span of underlying reward rates) and temporal variance (e.g., transient changes over time) increase the salience of these options and reward maximization [49,51,60,61]. The sham/low-variance condition had the same mean reward rate, but rates for each game were drawn from a high reward/low variance beta distribution (i.e., Beta(α = 30, β = 15), mean = 0.67; SD = 0.07; Fig. 1B). These settings were chosen based on simulations so that conditions only differed based on cross-sectional variance, while controlling for other elements of the reward environments, including pace of temporal variance (block horizon) and overall reward rate (high mean reward). All participants saw the same sets of rates within each condition, presented in randomized order.

### Assessment and computational modeling of RL performance

#### RL assessment task

A 3-arm MAB task (12 blocks of 16 trials; see Intervention section) with a neutral reward and variance environment (mean = 0.5; SD = 0.13) was used to assess reward learning and reward-maximizing behavior immediately prior to and after the training task. We chose such neutral environment parameters to provide more differentiation from both training conditions and because these experimental parameters of the MAB have been repeatedly tested in our prior work with evidence of reliable fitting of RL model parameters, including Bayesian RL models with strong model parameter recovery performance [34,35,62]. Each trial included a naturally jittered reaction time from decision signal (yellow token) to button press (decision phase) and the outcome receipt phase of 600 ms (i.e., from outcome presentation onset to next decision signal), followed by a 300 ms pause. A 6 s fixation cross appeared at the beginning and between each block. To encourage sufficient effort, participants were compensated a dollar amount proportional to their earned points in one randomly drawn game (ranging from $5 to $10), which was explained to them at the beginning of the experiment.

#### Computational modeling

To model RL performance, four models were considered, two learning models, each combined with one of two decision strategies. Learning models included: (a) a standard Rescorla–Wagner (RW) model [44,63] and (b) persistent prior RL (ppRL), an approximately Bayesian inference model [36,64] shown to predict human behavior in explorative reward paradigms more accurately than standard RL models such as RW [35,45,51]. Each learning model was tested in combination with either a standard or noisy Softmax function. Briefly, RW assumes that individuals update the estimated reward value for the chosen arm as a function of a prediction error term scaled by a learning rate parameter *η (*0 ≤ *η* ≤ 1), while estimates for the unchosen arms remain unchanged:

$${\hat{\mu }}_{k}^{t+1}={\hat{\mu }}_{k}^{t}+\eta {(R}^{t}-{\hat{\mu }}_{k}^{t}),\,{if}{D}^{t}=k$$

where ${\hat{\mu }}_{k}^{t}$ is the estimated reward value for arm k at trial t, R^t^ is the reward outcome obtained on trial t (i.e., *R* = 0 or *R* = 1), ${(R}^{t}-\,{\hat{\mu }}_{k}^{t}$) is the prediction error, and *D*^*t*^ is the decision made at trial t.

The ppRL model continuously injects a persistent prior term [36,64] in addition to updating reward expectations with the prediction error. Moreover, instead of a constant learning rate (for RW), ppRL uses a dynamic learning rate *λ*^*t*^, akin to a Kalman gain parameter [65], to update reward estimates for the chosen arm, while value estimates for the unchosen arms are updated solely by mixing in the persistent prior term:

$${\hat{\mu }}_{k}^{t+1}=\left(1-\gamma \right){\mu }_{0}+\gamma \,({\hat{\mu }}_{k}^{t}+{\lambda }^{t}{(R}^{t}-{\hat{\mu }}_{k}^{t})),\,{if}{D}^{t}=k$$

$${\hat{\mu }}_{k}^{t+1}=\left(1-\gamma \right){\mu }_{0}+\gamma \,{\hat{\mu }}_{k}^{t},\,{if}\,{D}^{t}\ne k$$

where parameter *γ (*0 ≤ *γ* ≤ 1) determines the degree to which reward estimates are mixed with a persistent prior value *μ*_*0*_ (initial belief) on each trial update, thus capturing the extent to which the underlying reward rates are estimated to be stationary. The dynamic learning rate *λ*^*t*^ is updated based on the uncertainty of the previous estimate ${\hat{\sigma }}_{k}^{2}$ (variance of the expected value estimate of the chosen arm) and the uncertainty in the “measurement” provided by each outcome, *σ*^*2*^_*D*_, an individual-level noise variance parameter (Supplemental Methods). Thus, prediction errors are dynamically weighted by precision in estimates rather than being weighted by a constant learning rate (as in RW).

Probability of choosing each arm was modeled as a standard or noisy Softmax function, which assumes choice probabilities for each arm to be normalized exponential functions of the reward estimates. For the ppRL model, the noisy Softmax decision rule is:

$$P\left({D}_{t}=k\right)=\frac{{e}^{b({\hat{\mu }}_{k}^{t}+\varepsilon {\hat{\sigma }}_{k}^{t})}}{\sum _{i}{e}^{b({\hat{\mu }}_{i}^{t}+\varepsilon {\hat{\sigma }}_{i}^{t})}}$$

where *b* is an inverse decision temperature parameter, which can be interpreted as the degree of reward maximization or reward sensitivity (see [66]), as it captures the extent to which the arm with highest reward estimate is chosen/exploited (with increasingly larger values indicating a greater tendency to select the arm estimated to be most rewarding, while values closer to zero indicating random selection/choice stochasticity, i.e., no influence of learned reward prediction on choice); here, *ε* is the decision noise parameter, and ${\hat{\sigma }}_{k}^{t}$ is the standard deviation of the estimated reward rate for arm k. For the RW model, ${\hat{\sigma }}_{k}^{t}$ is replaced by a novelty index initialized at 0 for each arm and is inversely related to the number of times each arm has been selected within each game (Supplementary Methods). The standard Softmax policy is a special case of the above equation when *ε* = 0, which was also tested as a simpler alternative strategy. Model fit and comparison strategy is described in Supplementary Fig. S1 and Table S1.

#### Group-level analyses

A sample size of 50 participants was deemed necessary to detect at least medium effect sizes for group differences (Cohen’s *d* > 0.5) with a statistical power of 80%. Levene’s test was used to determine homogeneity of variance. Linear mixed-effect models (LMEs) were used to assess the effect of training condition on pre- to post-training change in behavioral markers of interest, with both time (pre vs post) and training condition (active vs sham) included as fixed-effect predictors, and subject included as a random factor. For trial-level binary outcomes, (e.g., choice type), generalized LMEs were used with outcome modeled as a binomial distribution with a logit link, with trial included as an additional fixed-effect predictor. Given that condition groups differed as in baseline proportion of individuals with a current MDD diagnosis, this variable was controlled for by including it as a covariate in all reported analyses. Marginal effects for contrasts of interest were assessed for significant interactions and corrected for multiple comparisons with Tukey method. To inform future research, exploratory analyses examined mediating effects of target RL mechanisms engaged during training on treatment-related affective change (R:mediation; which uses a quasi-Bayesian Monte Carlo method with 1000 simulations to obtain bootstrapped 95% CIs on indirect effects).

### Neuroimaging analyses

#### fMRI acquisition and preprocessing

All images were acquired using 3.0-T Prisma scanner, including high-resolution T1-weighted anatomical images and functional T2-weighted blood oxygen level-dependent images (detailed scanning parameters and preprocessing steps in the Supplementary Methods).

#### Individual and group level analyses

For each participant, five regressors were generated. One regressor was time locked to decision onset and to the subject’s reaction time (decision phase). Four regressors were time locked to outcome presentation onset and to its duration, including two binary regressors coding for outcome type (reward and no reward) and two regressors for each outcome type that were parametrically modulated by trial-level RPE based on participants’ fitted model parameters. Voxel-wise LMEs testing the interaction of time x treatment condition were conducted to assess areas of treatment-related change in activation to each of the 5 individual-level regressors. As with behavioral analyses, each LME included MDD diagnosis as a covariate. To correct for multiple comparisons, we used a cluster-based thresholding method based on Monte-Carlo simulations implemented in the 3dClustSim function, which accounts for smoothing using a permutation test-based method. A minimum of 14 contiguous voxels was found to result in a corrected cluster-wise activation probability of p < 0.05 based on a voxel-wise a priori probability of p < 0.001.

### Sensitivity analyses

Sensitivity analyses examined robustness of main results as a function of anhedonic severity at inclusion (i.e., SHAPS score>7^1^, which may reflect a more severe anhedonic pathology). Another set of analyses controlled for a broader set of variables to capture complex clinical profile of MDD in our main analyses (i.e., including anxious arousal and anhedonic depression as covariates).

## Results

### Behavioral outcomes

#### Model-based performance

**Fig. 2:**
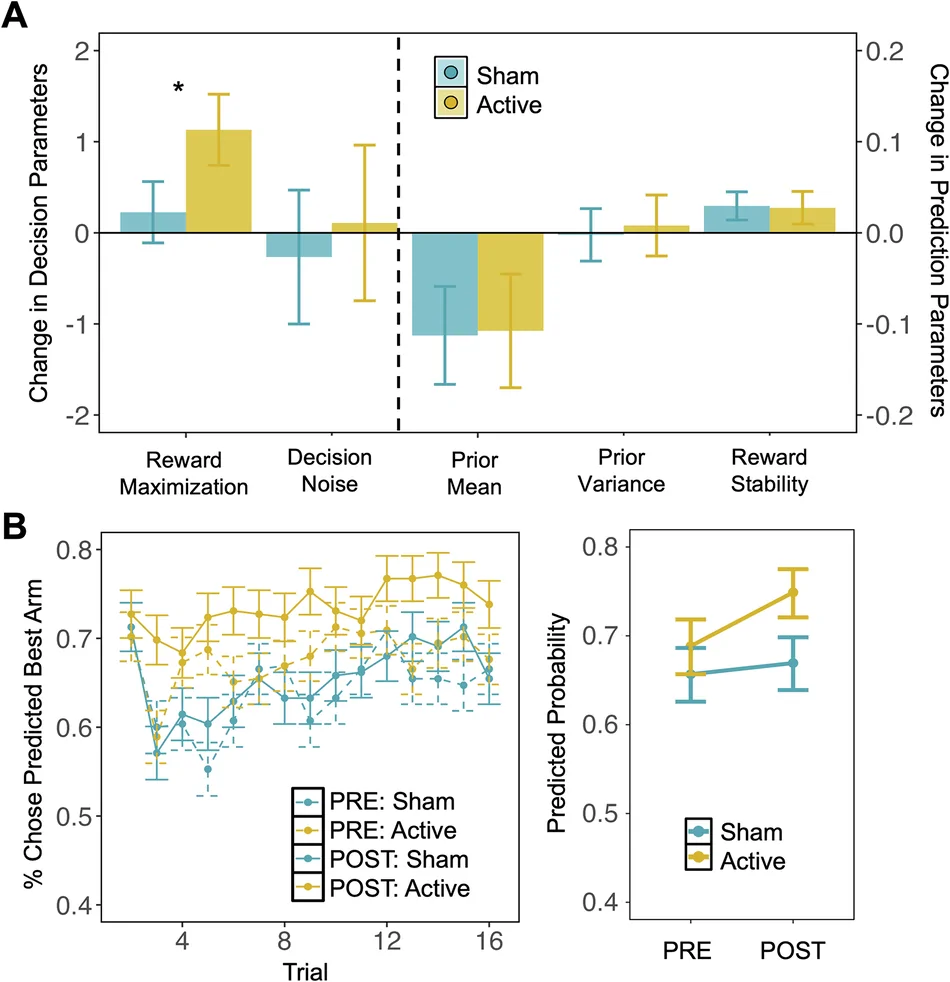
Training-related change in model-based behavior. **A** Change in model parameters by training condition. Decision parameters and associated scale are presented on the left side of dashed line: Reward Maximization (Softmax inverse decision temperature), Softmax decision noise. Parameters relating to learned reward predictions are presented on the right side of dashed line: mean and variance of the (initial) prior belief distribution, reward stability mixing parameter representing the degree to which reward rates are perceived as stable/non-volatile. The active condition was associated with a significant pre- to post-training increase in the reward maximization (inverse decision temperature) parameter (M = 1.13, *p* = 0.027; Cohen *d* = 0.99) relative to the sham condition (M = 0.23, *p* = 0.907; Cohen *d* = 0.19). **B** Change in learning-based reward maximizing choices. Left: Aggregate rates of choosing the arm predicted to be most rewarding as a function of time, training condition, and trial (throughout task learning horizon); Right: Marginal predicted probability for significant interaction of time and training condition. Across trials, participants in the active condition exhibited greater increase in the likelihood of making reward-maximizing choices (OR = 1.35, *p* < 0.001; Post-training predicted probability = 0.75, 95% CI = [0.72,0.78]) relative to those exposed to the sham condition (OR = 1.05, *p* = 0.646 ; Post-training predicted probability = 0.67, 95% CI = [0.64,0.70]); OR Odds Ratio.

As expected based on prior work [34–36,62], ppRL with a noisy Softmax decision strategy provided the best fit to participants decisions (Supplementary Table S1). Thus, the present results focus on pre- to post-training change in parameters related to this optimal model (for training performance and manipulation check, see Supplement). A significant time x training condition interaction was observed for the inverse decision temperature parameter *b* (F(1,50) = 4.8, *p* = 0.033), with greater increase in this reward maximization parameter in the active (M = 1.13, 95% CI = [0.36,1.87]) relative to sham group (M = 0.23, 95% CI = [−0.43,0.88]); Cohen *d* = 0.79). Training condition did not significantly shift other individual parameters, including decision noise, persistent prior mean and variance, perceived reward stationarity*;* (*ps* > 0.05; Fig. 2A). Training condition had a significant effect on learning-based reward maximization (χ^2^(1) = 12.2, *p* < 0.001). Specifically, a greater increase in individuals’ likelihood to choose options they predicted to be most rewarding was observed in the active (odds ratio/OR = 1.35, *p* < 0.001; post-training predicted probability = 0.75, 95% CI = [0.72,0.78]) relative to sham group (OR = 1.05, *p* = 0.646; post-training predicted probability = 0.67, 95% CI = [0.64,0.70]; Fig. 2B). Thus, as hypothesized, the active training condition promoted significantly greater increases in goal-directed reward maximization, reflecting an increased tendency to maximize reward outcomes based on internal predictions.

#### Model-agnostic behavior

**Fig. 3:**
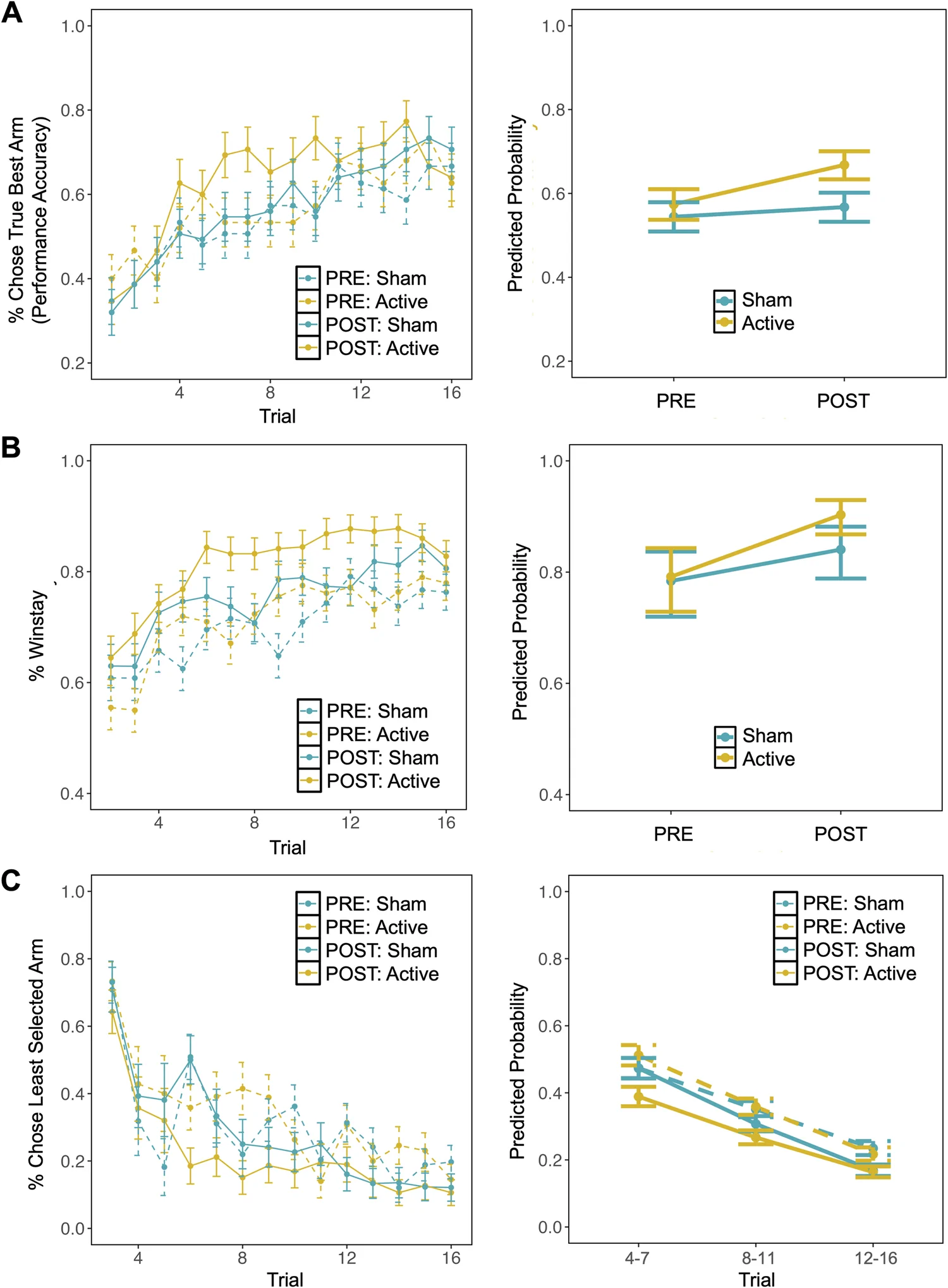
Model-agnostic behavioral performance as a function of trial, time (pre vs post-training) and training condition. **A** Performance accuracy, i.e., rate of choosing arm with highest true reward rate (based on experimentally set reward rates; minimum gap Δ ≥ 0.15). Left: Aggregate rates. Right: Marginal predicted probabilities for the significant interaction of time and training condition. Participants in the active condition showed an increase in the likelihood of choosing the best arm (OR = 1.49, *p* < 0.001; post-training predicted probability = 0.66, 95% CI = [0.63,0.70]), a pattern which was not apparent by the sham group (OR = 1.09, *p* = 0.763; post-training predicted probability = 0.57, 95% CI = [0.54,0.60]). **B** Winstay choices. Left: aggregate rates. Right: Marginal predicted probabilities for the significant interaction of time and training condition. Participants in the active condition showed a greater increase in the likelihood of selecting the same arm following a win, i.e., “winstay” (active group: OR = 2.61 *p* < 0.001; post-training marginal predicted probability = 0.91, 95% CI = [0.87,0.94]; sham group: OR = 1.41, *p* = 0.001; post-training marginal predicted probability = 0.84, 95% CI = [0.79,0.88]. **C** Rate of choosing least sampled arm (exploration). Left: Aggregate rates. Right: Marginal predicted probabilities for a significant time x training condition x trial interaction. Participants in the active condition showed an decrease in the likelihood of selecting the least sampled arm, which was apparent in middle portions of the game horizon, i.e., trials 4–7 (active group: OR = 0.48, *p* < 0.001; post-training marginal predicted probability = 0.35, 95% CI = [0.30,0.39]; sham group: OR = 1.11, *p* = 0.955; post-training marginal predicted probability = 0.49, 95% CI = [0.44,0.54]), trials 8–11 (active group: OR = 0.51, *p* < 0.001; post-training marginal predicted probability = 0.22, 95% CI = [0.19,0.24]; sham group: OR = 0.81, *p* = 0.341; post-training marginal predicted probability = 0.27, 95% CI = [0.24,0.29]). OR Odds Ratio.

The pattern of learning-based reward maximization increase in the active training group was accompanied by treatment effects on model-agnostic behavior. Consistent with greater shift towards reward maximization, the active group exhibited greater pre- to post-training increases in performance accuracy, i.e., choosing the arm with true highest rate (χ^2^(1) = 4.9,*p* = 0.026; active: OR = 1.49,*p* < 0.001; sham: OR = 1.09,*p* = 0.763; Fig. 3A) as well as greater increases in win-stay choices (χ^2^(1) = 24.1,*p* < 0.001; active: OR = 2.61,*p* < 0.001; sham: OR = 1.41,*p* = 0.001; Fig. 3B). Moreover, the active group showed greater pre- to post-training decreases in choosing the least sampled option, which was moderated by trial (χ^2^(1) = 6.2,*p* = 0.013). Specifically, this greater decrease in the active group was most apparent in the middle of the game horizon, i.e., trials 4–7 (active: OR = 0.48, *p* < 0.001; sham: OR = 1.11,*p* = 0.955) and trials 8–11 (active: OR = 0.51, *p* < 0.001; sham: OR = 0.81, *p* = 0.341; Fig. 3C). Training condition did not significantly modulate pre- to post-training change in rates of lose-shift choices (*ps* > 0.05; Supplementary Fig. S2). Overall, these results are consistent with a greater shift in the active group from the need to explore reward options towards more efficient reward maximization.

### Affective change

**Fig. 4:**
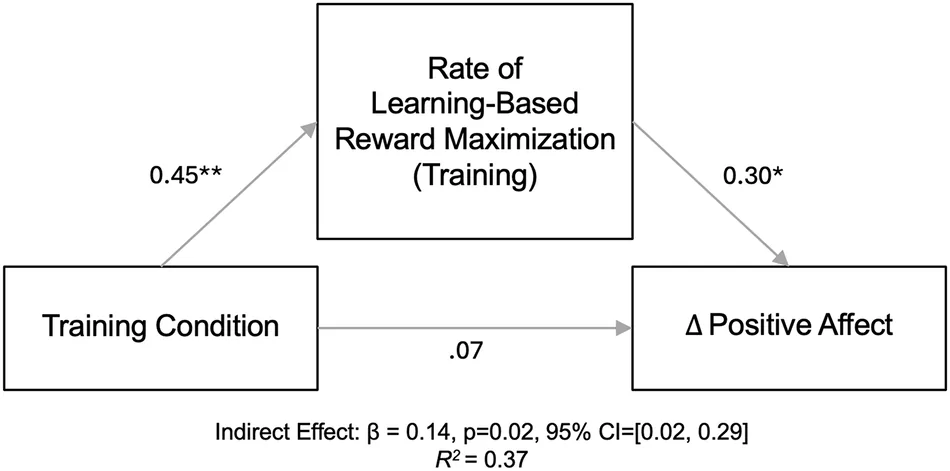
Mediation of training eff ect on positive affect. Learning-based reward maximization during training mediated the impact of the active training condition on positive aff ect. The active condition was associated with greater rate of choosing the option predicted to be most rewarding during training, which in turn predicted larger pre- to post-training increase in positive aff ect (ps<.05). A statistically signifi cant indirect eff ect between training condition and pre- to post-training change in positive aff ect was observed via learning-based reward maximizing performance during training (b=0.14, p=0.018, 95% CI=[0.02, 0.29].

A differential impact of training condition on affective change based on affective valence (positive vs negative) was observed after controlling for baseline affect and MDD status (F(1,90) = 3.96, *p* = 0.049). Specifically, greater increase in positive affect was seen in the active (M = 1.18, 95% CI = [−0.61,2.97]) relative to sham (M = −1.55, 95% CI = [−3.35,0.23; Cohen *d* = 0.63) condition, whereas training condition did not predict change in negative affect (*p* > 0.05). Moreover, target engagement during training was differentially related to affective change based on affective valence (F(1,90) = 4.1, *p* = 0.046), with a significant positive relationship between learning-based reward maximization during training and increase in positive affect (β = 0.35, p = 0.008) but not negative affect (*p* > 0.05). Based on these findings, an exploratory mediation analysis was conducted to assess whether learning-based reward maximization during training mediated this impact of treatment on positive affect. The active condition was associated with greater rate of choosing the option predicted to be most rewarding during training (β = 0.45,p = 0.001, 95% CI = [0.20,0.71]), which in turn predicted larger pre- to post-training increase in positive affect (β = 0.30, p = 0.033, 95% CI = [0.03,0.56]). A statistically significant indirect effect between training condition and pre- to post-training change in positive affect was observed via learning-based reward maximizing performance during training (β = 0.14, p = 0.018, 95% CI = [0.02, 0.29]; Fig. 4).

### Neural outcomes

**Fig. 5:**
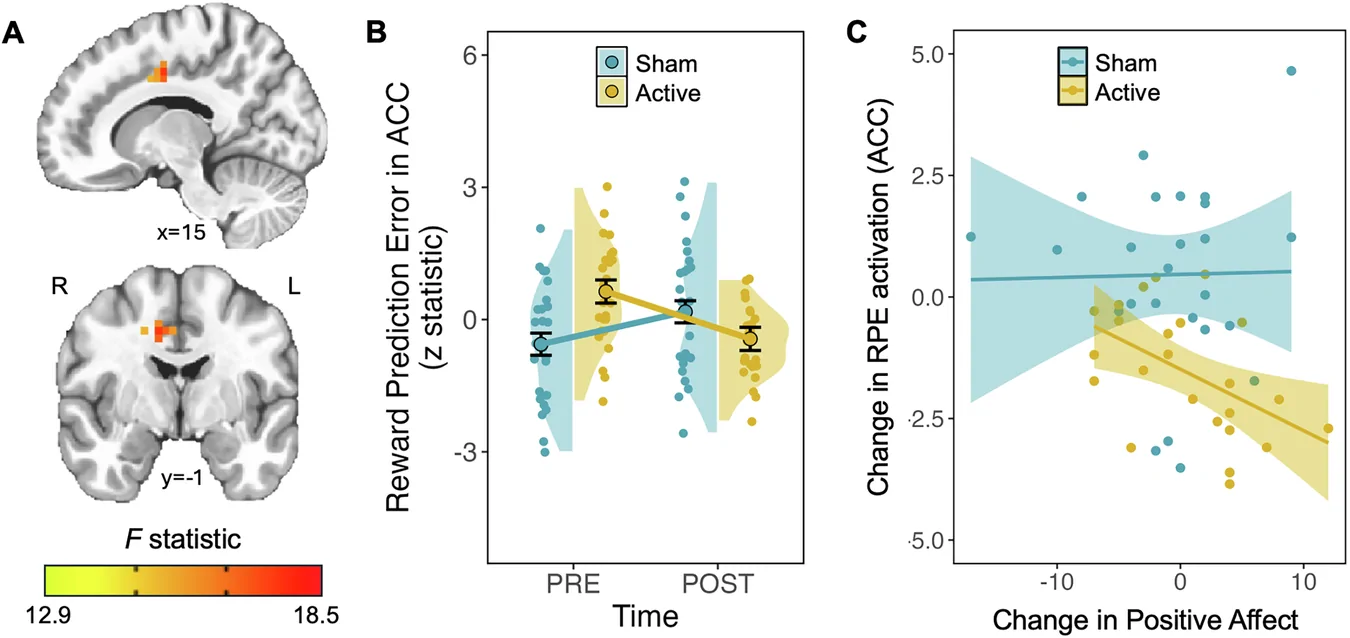
Training-related change neural activation. **A** Sagittal and coronal views of the brain illustrating a cluster in the dorsal anterior cingulate cortex (dACC; volume of 15 4 mm × 4 mm × 4 mm voxels/960 mm^3^; peak voxel MNI coordinates: 12, − 1,41) showing a significant time x training condition interaction in neural activation to model-based prediction errors during reward outcomes, i.e., reward prediction error (RPE); the color bar and color clusters represents F values**. B** Plot of individual’s average RPE activation and associated marginal means as a function of time and training condition. A significant decrease in RPE activation was observed in the active group (Cohen *d* = −1.04, *p* < 0.001), while the opposite pattern was seen in the sham group, albeit not statistically significant after Tukey correction (*d* = 0.71, *p* = 0.071). **C** Training condition significantly moderated the relationship between pre- to post-training change in RPE activation in this ACC cluster and pre- to post-training change in positive affect. A significant negative relationship was observed in the active group (β = −0.63, 95% CI = [−0.14,−1.11]), but not in the sham group (0.04, 95% CI = [−0.29,0.36]).

Whole brain analyses revealed one cluster in the ACC in which activation was associated with a significant time (pre- vs post-training) by condition (active vs sham) interaction associated with RPE in response to reward outcomes (BA 24; 960mm^3^; F-statistic=13.6, family-wise error *p* < 0.05; peak voxel MNI-coordinates: 12, −1,41). In this area, greater reduction in RPE activation was observed in the active relative to sham group (Fig. 5A-B). In follow-up exploratory analyses, training condition also moderated the relationship between change in RPE activation in this region (i.e., extracted individuals’ beta weights for the above contrast) and change in positive affect (F(1,44) = 4.96, *p* = 0.031). Specifically, greater pre- to post-training reduction in RPE activation was associated with greater increase in positive affect in the active group (β = −0.63, 95% CI = [−0.14,−1.11], but not the sham group (0.04, 95% CI = [−0.29,0.36]; Fig. 5C). No other cluster of neural activation survived multiple comparison correction for other contrasts of interest, including time x condition interaction predicting prediction error during non-reward outcomes, reward anticipation, or for non-computationally weighted regressors.

### Sensitivity analyses

Main results remained significant after controlling for a broader set of clinical indices associated with MDD and they were robust to baseline anhedonic severity at inclusion (Supplementary Figs. S3–S5).

## Discussion

This study investigated the neurobehavioral effects of a novel, computationally informed cognitive training to explore its potential as a targeted intervention for reward sensitivity, given the prevalence of reward responsiveness impairment across psychiatric conditions with anhedonic pathology. In a double-blind 2-arm RCT, we show that a single session of an RL task tuned to engage participants to learn from a high-variance reward environment leads to significant increases in learning-based reward maximization among anhedonic individuals. Enhanced reward learning and sensitivity were observed at the behavioral and neural levels, along with preliminary evidence that such training target engagement may be associated with positive affect increase.

Relative to sham, the active condition was associated with greater pre- to post-training increases in learning-based reward maximization. This was evidenced by greater increases in a reward sensitivity parameter and a higher rate of choosing options predicted to be most rewarding (i.e., model-based performance). Importantly, this pattern was reflected by improvements in model-agnostic performance, including greater increase in performance accuracy (i.e., rate of choosing option with true highest rate) and reward exploitation (i.e., winstay behavior), as well faster reduction in exploration, which is overall consistent with more goal-directed reward maximization. Consistent with these findings, impairments in reward sensitivity have been linked to anhedonia in depressed and trauma-exposed individuals [34,35,39,67], with evidence of lower inverse temperature parameters (i.e., lower reward sensitivity) rather than abnormalities in learning rates (although anhedonia may impact learning rates for punishments [68]). Our findings suggest that our intervention may be able to specifically target and improve learning-based reward sensitivity deficits, in contrast to shifting other aspects of RL (e.g., learning rate). While studies of RL trainings are scarce, one recent study suggests that shifting learning rates is feasible, although, enhanced learning was only observed for negative rather than rewarding outcomes [69]. The present study suggests that RL-based trainings may also be useful to shift and optimize reward-maximizing behavior.

We also observed a double dissociation of training-related change in RPE activation in the dorsal ACC. Specifically, while the sham group showed a mild increase in RPE activation, the active group exhibited decreased RPE activation in this region. Moreover, this effect was specific to reward outcomes, whereas no training-related differences was observed for prediction error weighted activation to non-reward outcomes or non-model-based activation to reward outcomes. The ACC is robustly linked to RPE signaling, which reflects the degree of discrepancy between expected and observed outcome, which is in turn used to update predictions [37,38]. Thus, a post-training reduction in RPE activation could indicate more efficient learning [41], i.e., reduced neural surprise reflecting more accurate predictions, consistent with improved performance observed in the active group. Interestingly, reduction in RPE activation has been observed following cognitive behavioral treatments, including prolonged exposure [70] and mindfulness training [71,72]. Alternatively, the ACC has been specifically linked to monitoring outcome uncertainty/volatility in the environment [48,73] and supporting adaptative behavioral changes to reduce uncertainty [74], e.g., greater RPE activation in this region promotes shifting to more reward exploration and learning [52]. Thus, reduced RPE signaling in the ACC could reflect a reduction in perceived volatility of the reward environment in the active group. Given that the active intervention exposed participants to more variable rewards, it is plausible that this training could improve uncertainty minimization, which is aligned with a faster shift from exploration to exploitation/reward maximization (consistent with our behavioral results, Fig. 3C). This pattern of RPE activation change was not observed in the striatum, which may seem at odds with evidence of blunted RPE neural signals during reward learning in depression [40,41]. However, evidence of striatal RPE abnormalities in depression is less clear when model-based valuation is accounted for [40] and some computational studies have also failed to observe a link between anhedonia and RPE signaling amplitude cross-sectionally [41,75,76]. Nonetheless, given the absence of a healthy control group to assess RPE activations in comparison to clinically normative levels at baseline and the observed group differences in RPE activation at baseline (despite randomization), future studies are needed to further explore and unpack these findings.

Finally, greater increase in positive affect was observed in the active relative to sham condition, after controlling for baseline positive affect and MDD diagnosis status. This effect was mediated by the degree of learning-based reward maximization during training, which provides a preliminary link between our treatment target and affective change in the expected direction for improving anhedonia. However, caution is needed when interpreting these findings as within-group affective changes were minimal and the present study was not designed to assess training impact on clinical pathology at this stage. Mediation analyses were also exploratory given the sample size. Minimal affective change is not surprising in the context of a brief, single training session over the span of an hour. Nonetheless, the positive relationship between engagement in our target reward learning mechanism during training and increase in positive affect is intriguing and suggests that learning-based reward maximization may be a reasonable therapeutic target to improve positive affect and anhedonia. A neurocomputational intervention targeting this mechanism to boost patients’ propensity to learn and obtain rewards in the real world could presumably lead to improvements in mood via feedback loops of engaging in pursuing rewards, which remains to be empirically tested. Thus, an important next step would be to assess how large and long-lasting positive mood improvements could be, e.g., following multiple-session protocols of the proposed RL training, and whether additional context is needed to produce sustained affective changes. For instance, a multi-week version of this computerized training (e.g., 8–16 weekly sessions) could be developed, including a greater range of reward variance settings and reward stimuli (e.g., social, multi-sensory), and could be evaluated as a stand-alone treatment, as patients engage in their daily lives, or as an adjunctive modality to pharmacotherapy or psychotherapy treatments, such as CBT or BA. It would be particularly important to assess real-world long-term outcomes (e.g., via Emotional Momentary Assessment) during and weeks following end of treatment to gauge the true clinical potential of this intervention.

This study had several limitations. First, the sample size was relatively modest, although the study was adequately powered to detect at least medium effect sizes for individual-level measures. Secondly, while our assessment RL task had different experimental settings than those used for the training blocks (i.e., unlike training, assessment blocks had a neutral, moderate variance reward environment), the same MAB paradigm was used for both training and assessment. While an important first step in assessing training effects, this study was not designed to assess generalization of training to a broader range of RL settings, which should be targeted in follow-up research. For instance, it will be critical to test whether this protocol can shift behavior in similar RL paradigms but with different types of stimuli beyond monetary reinforcers (e.g., social cues such as faces, other sensory stimuli) as well as different types of environments (e.g., different temporal schedules of reward variance, fixed vs open horizon, greater number of options). Critically, it is not known if this training can generalize to real world settings and promote goal-directed reward-maximizing choices in daily life (e.g., hobbies, social interactions). Finally, while this study was a double-blind RCT and condition groups were overall similar in demographic and clinical baseline profiles, a notable exception was percentage of individuals with an MDD diagnosis (higher in the active group). The inclusion criteria pertaining to anhedonia may have also introduced heterogeneity, although groups did not differ in baseline levels of trait anhedonia (MASQ) and positive affect (PANAS; Table 1). All statistical analyses, including whole-brain analyses, controlled for MDD diagnosis, and sensitivity analyses confirmed that additional clinical covariates (e.g., anxiety and anhedonia) did not impact the pattern of findings.

In conclusion, the present study provides preliminary evidence that a novel computerized training paradigm rooted in a RL-based framework of reward responsiveness can boost reward-seeking behavior in anhedonic individuals. These effects were observed across a range of neurocomputational and behavioral markers. Additional research is needed to investigate the robustness of these effects on real-world behavior and long-term affective symptom severity. If clinical effects are established, this type of intervention could provide a scalable and low-cost treatment option to augment the effectiveness of current evidence-based treatments such as antidepressant therapy or psychotherapy.

## Supplementary information

Supplemental Material

## Data Availability

Data supporting the findings of this study are available from the corresponding author, upon reasonable request.

## Footnotes

1 While all participants had a baseline PANAS positive score <25, a subset of 15 (33%) also endorsed a SHAPS score>7 upon inclusion. Both active and sham groups had similar proportions of individuals with an inclusion SHAPS score >7 (χ^2^(1) = 0.09, *p* = 0.758; Active: 32%; Sham: 28%).

## Code availability

Code used for analyses is available upon request from the corresponding author.
