# Supplementary material for: Neurocomputational training to boost reward learning and sensitivity in anhedonic individuals: a transdiagnostic proof-of-concept trial
Source: Transl Psychiatry. 2026 Aug 3;16:600. doi: 10.1038/s41398-026-04355-1 (PMC13720024; doi:10.1038/s41398-026-04355-1)
Supplement: Supplementary file 1 [file 41398_2026_4355_MOESM1_ESM.pdf]

SUPPLEMENTARY MATERIAL

Neurocomputational Training to Boost Reward Learning and Sensitivity in Anhedonic Individuals: A Transdiagnostic Proof-of-Concept Trial.

Katia M. Harlé[1,2,3], Murray B. Stein[2], Alan N. Simmons[1,2,3,*], & Charles T. Taylor[2,*]

Supplementary Methods: Exclusion Criteria

Exclusion criteria were intended to ensure that participants could safely complete the procedures and to optimize generalizability while minimizing confounding interpretation of our findings: 1) lifetime diagnosis of bipolar or psychotic disorders; 2) recent moderate/severe alcohol or substance use disorder (past 3 months); 3) current active suicidal ideation; 4) concurrent pharmacological treatment most theoretically likely to impact reward processing, e.g., anxiolytics, benzodiazepines, dopamine-modulating antidepressants (e.g., Bupropion), and antipsychotics, however stable doses of SSRIs/SNRIs (continued use for past 4 weeks or more) were allowed for generalizability and feasibility; 5) concurrent evidence-based psychotherapy (started within past 4 weeks); and 6) characteristics that compromise MRI safety (e.g., metal in body, claustrophobia, neurosurgery).

Supplementary Methods: Computational modeling of reward learning

Four models (2 types of learning models x 2 types of decision policies) were applied to the participants' trial-wise MAB choices and compared for model fit. These included two classes of learning models: a) a standard Rescorla–Wagner (RW) model [1, 2] and b) a persistent prior RL (ppRL) model, which closely approximates a Bayesian inference RL model, i.e., the Dynamic Belief Model/DBM [3, 4] and has been shown to better predict human behavior in explorative reward paradigms relative standard RL models such as RW [5-7]. Each learning model was tested in combination with one of two types of decision strategy, a standard or noisy Softmax function (see below).

<u>Learning models:</u> Briefly, RW assumes that individuals update reward expectations for the chosen arm as a function of prediction errors scaled by a learning rate η parameter (constant at the individual level; $0 \leq \eta \leq 1$), while estimates for the unchosen arms remain unchanged:

$$\hat{\mu}_k^{t+1} = \hat{\mu}_k^t + \eta\,(R^t - \hat{\mu}_k^t),\ \ if\ D^t = k$$

$$\hat{\mu}_k^{t+1} = \hat{\mu}_k^t,\ \ if\ D^t \neq k$$

where $\hat{\mu}_k^t$ is the estimated reward for arm k on the previous trial t, R^t is the reward outcome obtained on trial t (i.e., R=0 or R=1), and $(R^t - \hat{\mu}_k^{t-})$ is the prediction error.

The ppRL model continuously injects a persistent prior term [3, 4] in addition to updating reward expectations with the prediction error. Thus, reward estimates are updated at each trial as a mixture of a prior mean value, the initial mean reward expectation, and the previous outcome. Rather than a constant learning rate, the ppRL model uses a dynamic learning rate λ^t , which is akin to a Kalman gain parameter [8]:

$$\hat{\mu}_k^{t+1} = (1-\gamma)\mu_0 + \gamma\,(\hat{\mu}_k^t + \lambda^t(R^t - \hat{\mu}_k^t)),\ \ if\ D^t = k$$

$$\hat{\mu}_k^{t+1} = (1-\gamma)\mu_0 + \gamma\,\hat{\mu}_k^t,\ \ if\ D^t \neq k$$

where parameter γ ($0 \leq \gamma \leq 1$) determines the degree to which reward estimates are mixed with a persistent prior value μ_0 (equivalent to the mean of the re-sampling fixed prior distribution in DBM) on each trial update. Thus γ captures the extent to which the underlying reward rates are estimated to be stationary, with γ=1 being as special case equivalent to a fixed belief assumption (i.e., no injection of persistent prior belief). In the ppRL model, the expected reward of unchosen arms is also updated by mixing the previous expected value with the persistent prior term. Following a Kalman gain process, the dynamic learning rate λ^t is updated on each trial based on the uncertainty of the previous estimate and the uncertainty in the "measurement" provided by each outcome:

$$\lambda^t = \frac{\hat{\sigma}_t^2}{(\hat{\sigma}_t^2 + \sigma_D^2)},$$

$$\hat{\sigma}_{t+1}^2 = \gamma^2(1 - \lambda^t)\hat{\sigma}_t^2 + \sigma_D^2$$

where $\hat{\sigma}_t^2$ is the variance of the expected reward estimate of *the chosen arm* at trial t, σ^2_D is an individually fitted noise variance parameter, γ is the mixing parameter. Thus, prediction errors are dynamically weighted by precision in estimates rather than being weighted by a fixed learning rate (as for the RW model).

<u>Decision policies:</u> In both RW and ppRL models, probability of choosing each arm was modeled as a standard or noisy Softmax function, which assumes choice probabilities for each arm to be normalized exponential functions of the reward estimates. The standard/pure Softmax function is:

$$P\ (D_t = k) = \frac{e^{b(\hat{\mu}_k^t)}}{\sum_i e^{b(\hat{\mu}_i^t)}}$$

where *b* is an inverse decision temperature parameter, which captures the degree of reward maximization, i.e., the extent to which the arm with highest reward estimate is likely to be selected and $\hat{\mu}_k^t$ is the reward estimate for arm k.

The noisy Softmax policy adds a weighted uncertainty term to the reward value estimates. For the ppRL model, the noisy Softmax decision policy models the probability of choosing each arm based on the estimated mean reward of each arm and the standard deviation of the estimate, weighted by a decision noise parameter (capturing the uncertainty sensitivity applied to mean reward estimates):

$$P\ (D_t = k) = \frac{e^{b(\hat{\mu}_k^t + \varepsilon\hat{\sigma}_k^t)}}{\sum_i e^{b(\hat{\mu}_i^t + \varepsilon\hat{\sigma}_i^t)}}$$

where ε is the uncertainty sensitivity parameter and $\hat{\sigma}_k^t$ is the standard deviation of the estimated reward rate for arm k. For the RW model, $\hat{\sigma}_k^t$ was replaced by a novelty index initialized at 0 for each arm and representing the negative of the number of times each arm has been selected in each game (i.e., also capturing a degree of uncertainty for a given arm reward prediction by counting the observations on each option; see [9]).

$$I_k^t = -\sum_{t'=1}^{t} I_{D_{t'}=k}$$

Note that when ε = 0, the decision policy reduces to the pure, non-noisy Softmax strategy.

Supplementary Methods: Model Fit and Comparison Strategy

Models were fit using hierarchical Bayesian estimation with the RStan interface of the Stan language [10], which uses a Hamiltonian Monte Carlo method. Up to 5 individual-level model parameters were fitted for ppRL, including 2 decision parameters (*b* and ε, representing reward maximization and decision noise, respectively) and 3 prediction related parameters (μ_0, σ^2_D, and γ, representing the initial reward expectation mean and variance, as well as perceived reward stationarity, respectively). Similarly, up to 4 model parameters were fitted for RW, including 2 decision parameters (*b* and ε) and 2 prediction-related parameters (μ_0 and learning rate η). Each class of models were tested with an individually fitted ε or a fixed $\varepsilon = 0$ (standard Softmax function). Model comparison and determination of the best fitting model was performed using leave-one-out cross-validation [11]. Individually fitted parameters and associated trial-wise reward prediction errors (RPEs) based on the winning model were used in subsequent behavioral and neural analyses. Model recovery analyses suggested a high degree of reliability for parameter estimates, with R^2 between true and recovered parameters >0.80 (see Supplementary Figure S1 and Supplementary Table S1).

Supplementary Figure S1: Simulation and Parameter Recovery

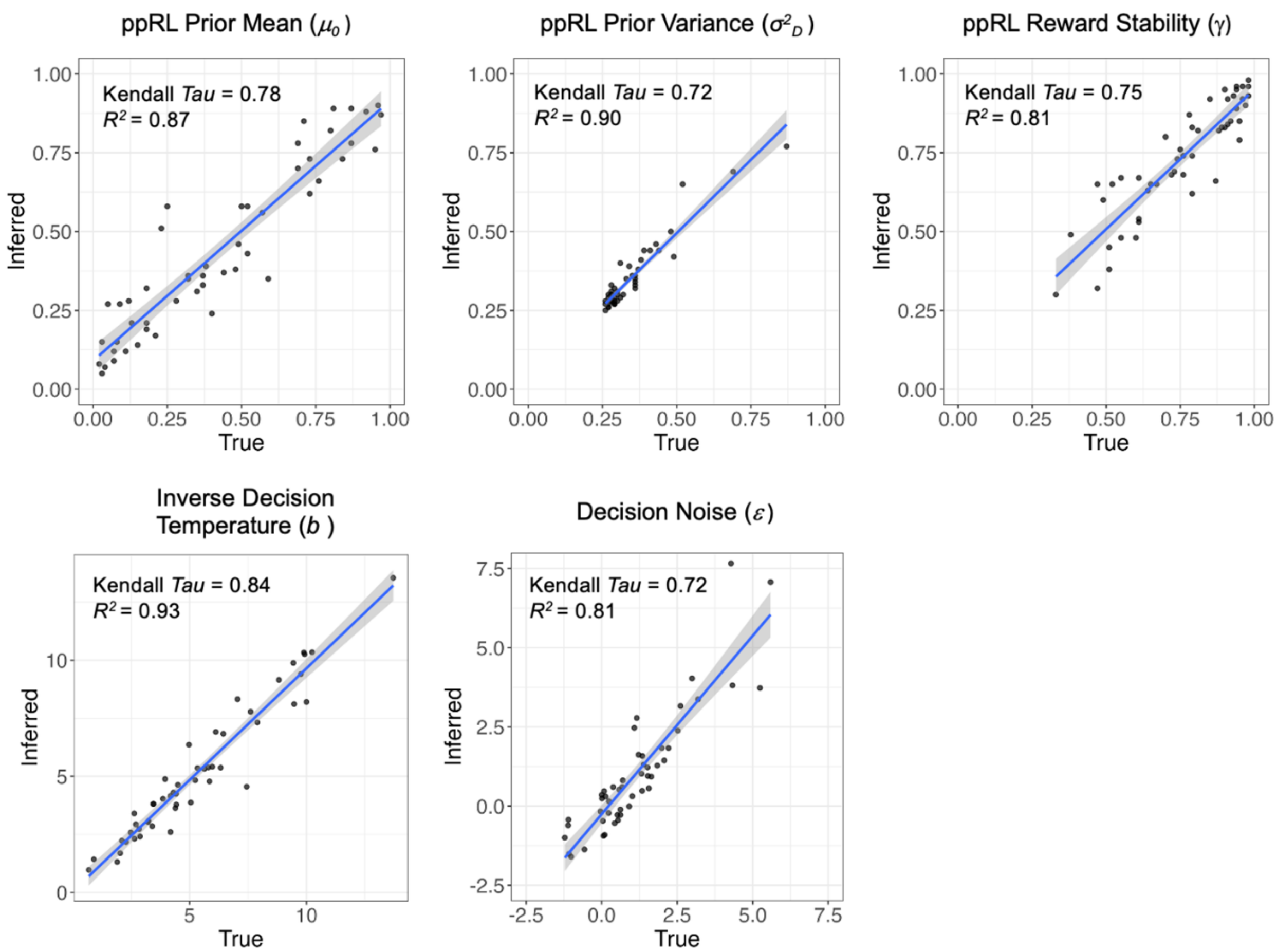

Note: Choice behavior was simulated for each of the parameter values estimated from our 50 participants at baseline (pre-training). Generated parameter values are on the x axis, and recovered values are on the y axis for ppRL fixed prior mean (top left) and variance (top center), ppRL reward stability parameter (top right), as well as Softmax inverse temperature parameter (bottom left) and decision noise (bottom center). Kendal Tau correlations ranged from 0.72 to 0.84 ($0.81 < R^2 < 0.93$).

Supplementary Table S1: Model Fit and Comparison Results

Model	Parameters	LOOIC	ELPD	ELPD SE	95% CI
ppRL – Noisy Softmax	μ_0, σ^2_D, γ, b, ε	14618	0	0	
RW – Noisy Softmax/ 2 η	μ_0, η_p, η_n, b, ε	14786	-97.6	28.1	[-140.3, -31.5]
RW – Noisy Softmax	μ_0, η, b, ε	14852	-128.8	28.1	[-171.3, -62.9]
ppRL – Standard Softmax	μ_0, σ^2_D, γ, b	14871	-138.5	13.5	[-152.1, -101.3]
RW – Standard Softmax	μ_0, η, b	14922	-163.6	29.1	[-207.8, -95.8]

Note: RW=Rescorla-Wagner model; ppRL=persistent prior reinforcement learning model; LOOIC=leave-one-out information criterion; SE=standard error; ELPD=Expected Log Pointwise Predictive Density (difference in expected log predictive density between upper model and current model); CI=Confidence Interval.

Supplementary Methods: Image Acquisition and Preprocessing

Scanner and Image Acquisition:

Participants were scanned in a 3 T Siemens Prisma scanner with an 32-channel head array coil. The scanning session included a three-plane scout scan and a sagittally acquired spoiled gradient recalled (SPGR) sequence to acquire T1-weighted high-resolution structural images (FOV 240 mm; matrix: 256×256 acquisition matrix; 176 slices; slice thickness: 0.94 mm; TR=2,300ms, TE=2.3ms, flip angle: 8°, inversion time=900ms). Participant also underwent one T2*-weighted axially acquired echo-planar imaging (EPI) scan to measure blood oxygen level dependent (BOLD) signal while they completed the functional tasks (parameters: 4 mm×4mm×4 mm; 64×64 acquisition matrix, 30 axially acquired slices; slice thickness: 4mm; TR=1,700 ms, TE=30 ms, flip angle=80°). To complete the task, participants used an MRI-safe button box, which they held in their hand, while the task was visible to them via a projected screen on a mirror in the head coil.

fMRI Preprocessing:

Preprocessing, normalization to MNI coordinates, and subsequent fMRI analyses were conducted using Advanced Normalization Tools Software for R (ANTsR) and Analysis of Functional NeuroImages (AFNI) software (67). Preprocessing steps included despiking and removal of temporal outliers, field inhomogeneity correction, slice time correction, temporal whitening, motion correction and a CompCor component-based noise correction [60]. One participant (in High-Variance group) was excluded from neuroimaging analyses due to excessive motion (>20% of brain volumes censored based on framewise displacement ≥5 mm). Regressors were convolved with a canonical hemodynamic response function (HRF) and entered into a general linear model (GLM) to obtain normalized beta weights and associated z statistics.

Supplementary Results: Training Manipulation Check

As expected, higher predictive accuracy of our Bayesian RL model (i.e., per-trial likelihood) was observed in the active (0.38) relative to sham (0.32; *d*=0.63, *p*=0.024) group. The active condition was also associated with greater likelihood of choosing the best option based on learned expectations (OR=1.88, $\chi^2(1) = 4.3$, *p*=0.037) and greater inverse decision temperature parameter (capturing the degree of learning-based reward maximization when selecting options; *d*=0.60, *p*=0.031). Other individual model parameters did not differ across conditions (*ps*>.05). Relative to the sham condition, those in the active condition also exhibited greater dynamic learning rates, which captures the degree to which reward predictions are updated based on prediction errors (β =0.14, $\chi^2(1) = 4.2$, *p*=0.041. Overall, these results suggest that the active training promoted learning-based (i.e., goal-driven) reward maximization to a greater extent than the sham protocol.

Supplementary Figure S2: Loseshift Behavior

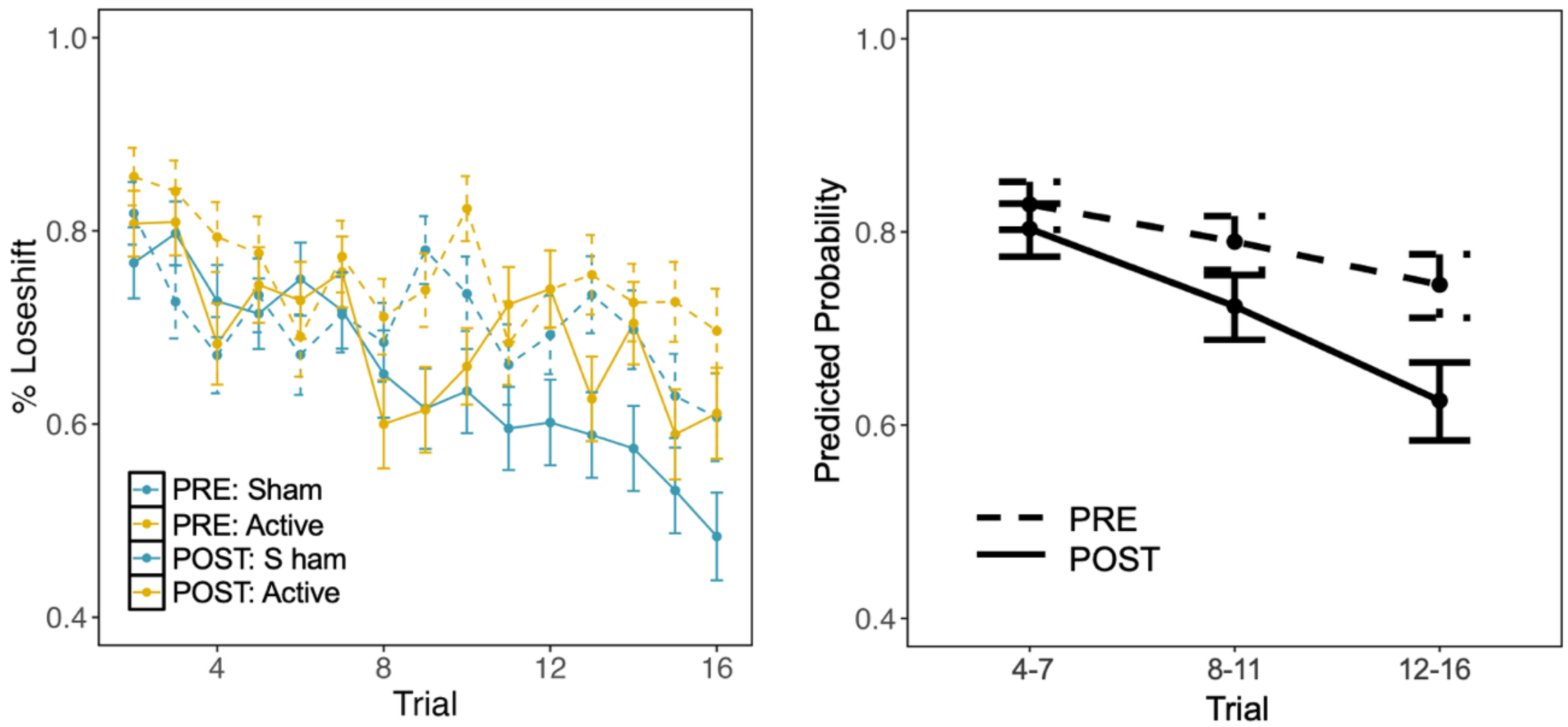

Note: Loseshift choices. Left: aggregate rates. Right: Marginal predicted probabilities for a significant interaction of time (pre- vs post-training) and trial ($\chi^2(1) = 6.2$, p=0.013). Likelihood of selecting a different arm following a no-reward outcome, i.e., "loseshift", was lower in the second half of the game, including trials 8-11 (pre-training marginal predicted probability=0.79, 95% CI=[0.76,0.81]; post-training marginal predicted probability=0.72, 95% CI=[0.69,0.75]), and trials 12-16 (pre-training marginal predicted probability=0.75, 95% CI=[0.71,0.78]; post-training marginal predicted probability =0.75, 95% CI=[0.71,0.78]). Training condition was did not significantly moderate loseshift likelihood (*ps*>.05). OR=Odds Ratio.

Supplementary Figure S3: Impact of SHAPS-Based Anhedonia Inclusion on Learning-Based Reward Maximization Outcomes

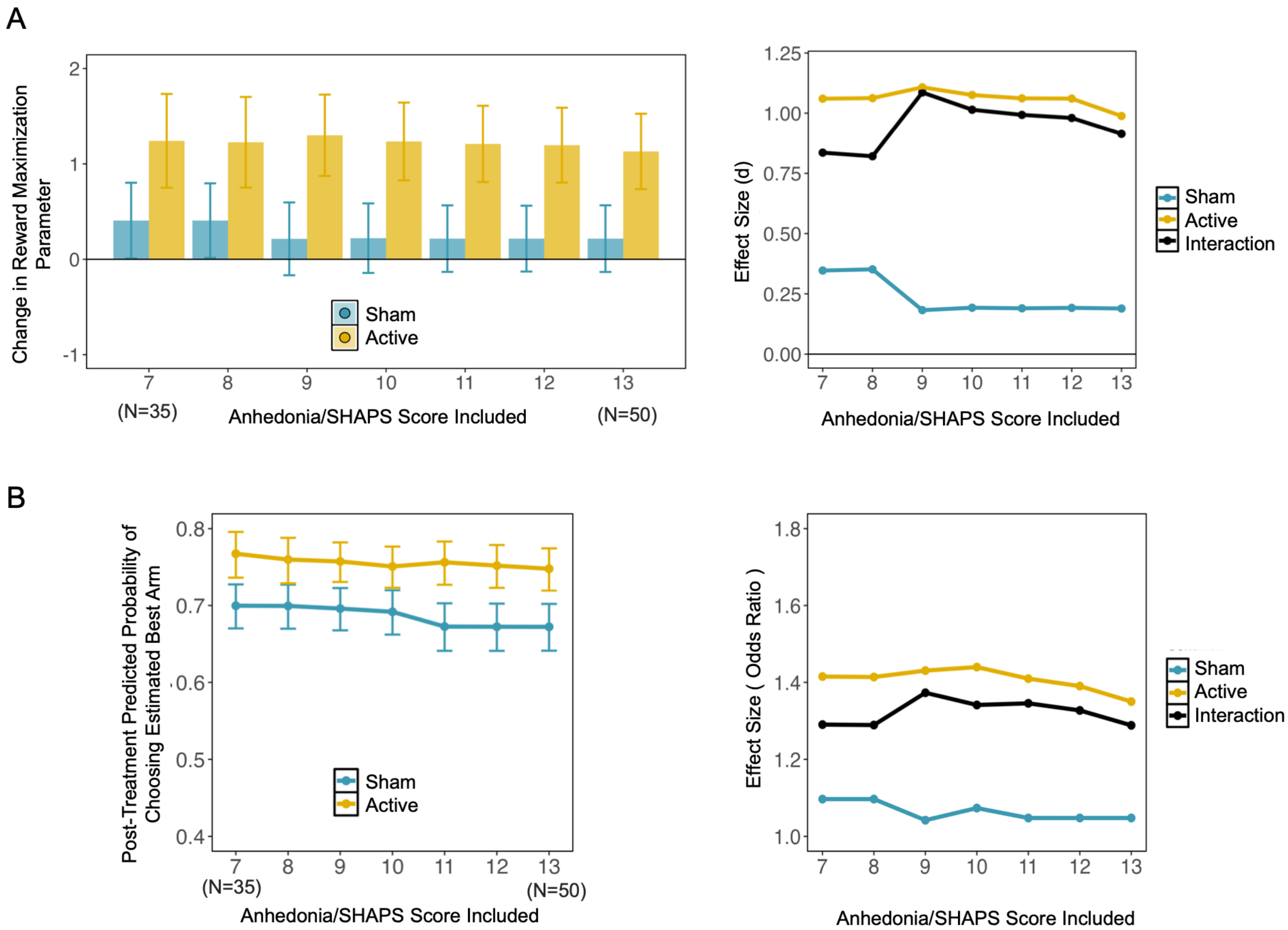

<u>Note:</u> **A.** Change in Softmax reward maximization parameter as a function of training condition. Left: when a subset of participants with an anhedonia SHAPS-based score >7 were excluded (N=35), greater pre- to post-training increase in this parameter (Softmax inverse decision temperature) was still observed in the active relative to the sham group (*d*=0.83 ; Active: M=1.24, Cohen *d*=1.06, 95% CI=[1.92,0.19]; Sham: M=0.24, *d*=0.34, 95% CI=[-0.33,1.03]). The same pattern was observed as more of these participants were added back in the total sample (up to the full sample N=50); Right: effect sizes associated with within-group pre-to-post training change and the time x group interaction within the same subsamples. **B.** Marginal predicted probability for the likelihood to choose the arm with estimated highest rate (learning-based reward maximization). Left: when a subset of participants with an anhedonia SHAPS-based score >7 were excluded (N=35), greater pre- to post-training increase in the likelihood of making reward-maximizing choices was observed in the active condition (odds ratio=1.42; Post-training predicted probability = 0.77, 95% CI=[0.74,0.79]) relative to those exposed to the sham condition (odds ratio=1.09; Post-training predicted probability = 0.70, 95% CI=[0.67,0.73]). The same pattern was observed as more of these participants were added back in the total sample (up to the full sample N=50); Right: effect sizes (odds ratio) associated with within-group pre-to-post training change as well as the time x group interaction within the same subsamples.

Supplementary Figure S4: Impact of SHAPS-Based Anhedonia Inclusion on Positive Affect and Reward Prediction Error (RPE) Activation in the ACC.

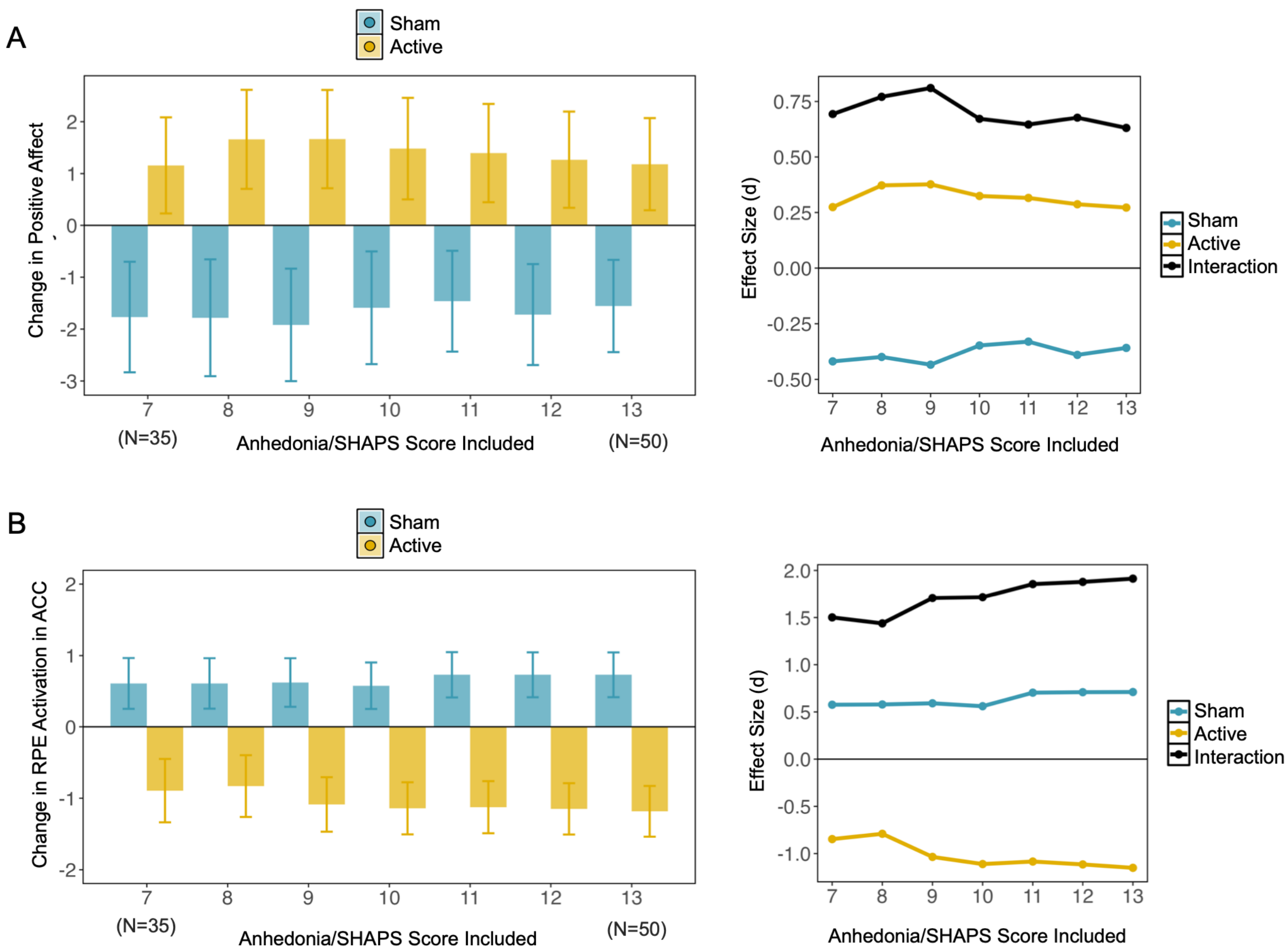

<u>Note:</u> **A.** Change in positive affect as a function of training condition. Left: When a subset of participants with an anhedonia SHAPS-based score >7 were excluded (N=35), greater pre- to post-training increase in positive affect was still observed in the active relative to the sham group (*d*=0.64; Active: M=+1.16, 95% CI=[-0.72, 3.04]; Sham: M=-1.77, 95% CI=[-3.93, 0.39]). The same pattern was observed as more of these participants were added back in the total sample (up to the full sample N=50); Right: effect sizes associated with within-group pre-to-post training change and the time x group interaction within the same subsamples. **B.** Marginal means of ACC cluster activation as a function of training condition. Left: when a subset of participants with an anhedonia SHAPS-based score >7 were excluded (N=35), a significant decrease in reward prediction error (RPE) activation in the ACC was observed in the active relative to sham group (*d*=1.50; Active: M=-0.85, 95% CI=[-1.68, -0.02]; Sham: M=0.57, 95% CI=[-0.11, 1.26]). The same pattern was observed as more of these participants were added back in the total sample (up to the full sample N=50); Right: effect sizes (odds ratio) associated with within-group pre-to-post training change as well as the time x group interaction within the same subsamples. ACC=Anterior Cingulate Cortex.

Supplementary Figure S5: Main analyses controlling for anxious arousal and anhedonic depression.

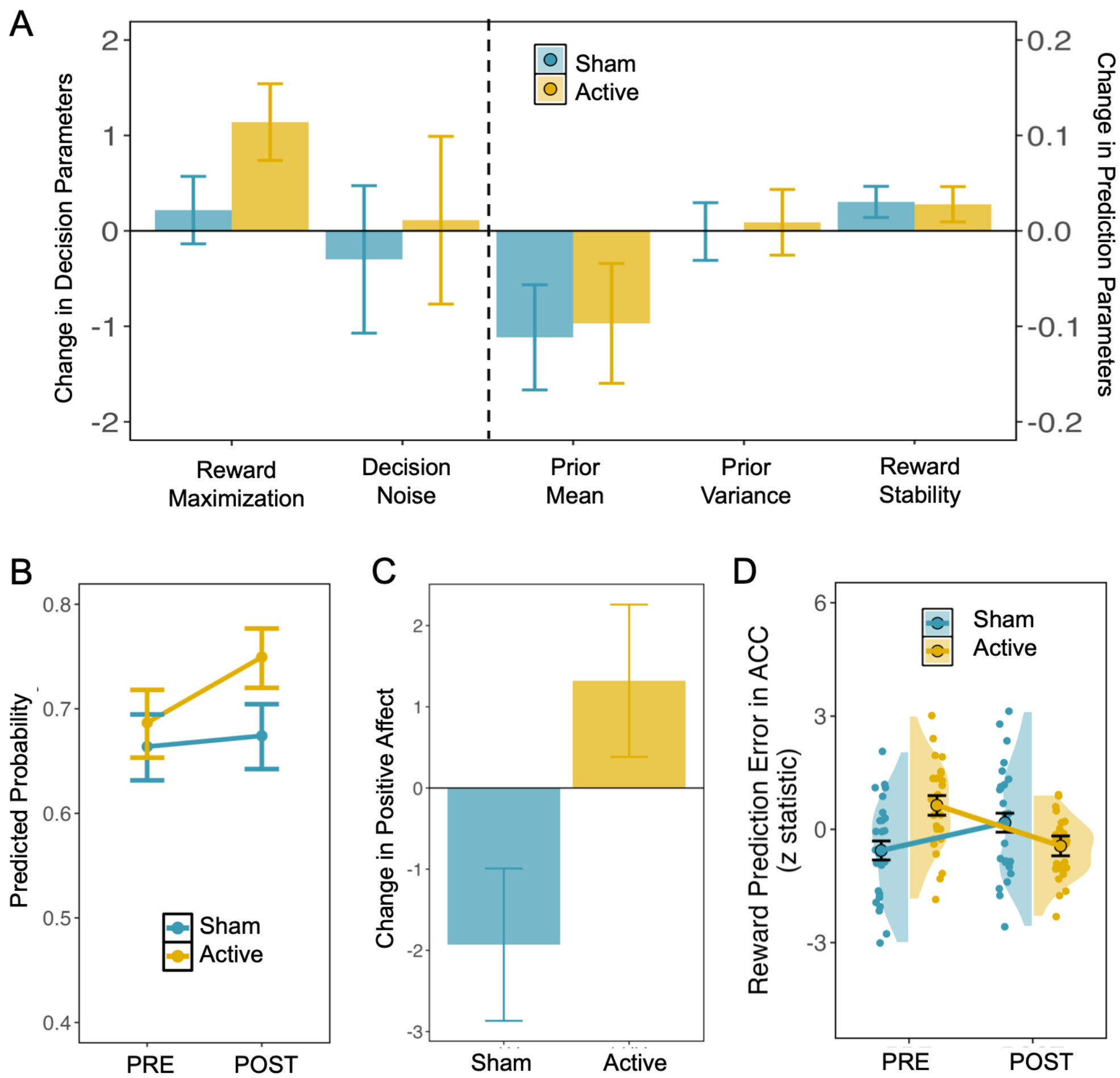

Note: Training condition on main outcomes were unchanged after controlling for anxious arousal and anhedonic depression severity, with similar or slightly larger effect sizes. **A.** Change in model parameters by training condition. The active condition was associated with a significant pre- to post-training increase in the reward maximization parameter (Softmax inverse decision temperature; M=1.14, *p*=.032; Cohen *d*=0.99) relative to the sham condition (M=0.22, *p*=.927; *d*=0.19). Training condition did not significantly impact other parameters. **B.** Marginal predicted probability for the likelihood to choose the arm with estimated highest rate (learning-based reward maximization). Across trials, participants in the active condition exhibited greater increase in the likelihood of making reward-maximizing choices (odds ratio=1.36, *p*<.001; Post-training predicted probability = 0.75, 95% CI=[0.72,0.78]) relative to those exposed to the sham condition (odds ratio=1.05, *p*=.652 ; Post-training predicted probability = 0.67, 95% CI=[0.64,0.70]). **C.** A differential impact of training condition on affective change based on affective valence (positive vs negative) was observed (F(1,84)=4.57, p=0.035; *d*=0.74); greater increase in positive affect in the active condition (M=+1.32, 95% CI=[-0.57,3.21]) relative to sham group (M=-1.93, 95% CI=[-3.82,-0.03]), whereas training condition did not predict change in negative affect (p>.05). **D.** Marginal means of ACC cluster activation as a function of time and training condition. A significant decrease in RPE activation was observed in the active group (*d*=-1.01, *p*<.001), while the opposite pattern was seen in the sham group, albeit not statistically significant after Tukey correction (*d*=0.73, *p*=.071). ACC=Anterior Cingulate Cortex.

References

[1] Rescorla RA, Wagner AR. A theory of Pavlovian conditioning: Variations in the effectiveness of reinforcement and nonreinforcement. Classical conditioning II: Current research and theory. New York: Appleton-Century-Crofts; 1972, p. 64-99.

[2] Niv Y, Montague PR. Theoretical and empirical studies of learning. Neuroeconomics. Elsevier; 2009, p. 331-51.

[3] Guo D, Yu AJ. Revisiting the Role of Uncertainty-Driven Exploration in a (Perceived) Non-Stationary World. *CogSci... Annual Conference of the Cognitive Science Society. Cognitive Science Society (US). Conference.* 43. NIH Public Access; 2021:2045.

[4] Ryali C, Reddy G, Yu AJ. Demystifying excessively volatile human learning: A Bayesian persistent prior and a neural approximation. Adv Neural Inf Process Syst 2018;31.

[5] Daw ND, O'Doherty JP, Dayan P, Seymour B, Dolan RJ. Cortical substrates for exploratory decisions in humans. Nature 2006;441(7095):876-9. https://doi.org/10.1038/nature04766.

[6] Harlé KM, Guo D, Zhang S, Paulus MP, Yu AJ. Anhedonia and anxiety underlying depressive symptomatology have distinct effects on reward-based decision-making. PLoS One 2017;12(10):e0186473.

[7] Guo D, Yu A. Why so gloomy? A Bayesian explanation of human pessimism bias in the multi-armed bandit task. *Advances in Neural Information Processing Systems*. 2018:5176-85.

[8] Simon D. Optimal state estimation: Kalman, H infinity, and nonlinear approaches. Hoboken, NJ: John Wiley & Sons; 2006.

[9] Cogliati Dezza I, Yu AJ, Cleeremans A, Alexander W. Learning the value of information and reward over time when solving exploration-exploitation problems. Sci Rep 2017;7(1):16919.

[10] Stan Development Team. RStan: the R interface to Stan. 2018.

[11] Vehtari A, Gelman A, Gabry J. Practical Bayesian model evaluation using leave-one-out cross-validation and WAIC. Statistics and Computing 2017;27(5):1413-32. https://doi.org/10.1007/s11222-016-9696-4.
